# Differential expression of circulating exosomal microRNAs in refractory intracranial atherosclerosis associated with antiangiogenesis

**DOI:** 10.1038/s41598-019-54542-y

**Published:** 2019-12-19

**Authors:** Hao Jiang, Juan F. Toscano, Shlee S. Song, Konrad H. Schlick, Oana M. Dumitrascu, Jianwei Pan, Patrick D. Lyden, Jeffrey L. Saver, Nestor R. Gonzalez

**Affiliations:** 1 Department of Neurosurgery, Neurovascular Laboratory, Cedars-Sinai Medical Center, Los Angeles, CA USA; 2 Department of Neurosurgery, The First Affiliated Hospital of Zhejiang University, School of Medicine, Hangzhou, China; 3 Department of Neurology, Cedars-Sinai Medical Center, Los Angeles, CA USA; 4 Department of Neurology, University of California, Los Angeles, Los Angeles, CA USA

**Keywords:** Prognostic markers, Stroke

## Abstract

Intracranial atherosclerotic disease (ICAD) is a common cause of stroke with high rates of ischemic recurrence. We aimed to investigate the role of circulating exosomal microRNAs (e-miRNAs) in recurrent ischemic events in ICAD. Consecutive patients with severe ICAD undergoing intensive medical management (IMM) were prospectively enrolled. Those with recurrent ischemic events despite IMM during 6-month follow up were algorithmically matched to IMM responders. Baseline blood e-miRNA expression levels of the matched patients were measured using next generation sequencing. A total of 122 e-miRNAs were isolated from blood samples of 10 non-responders and 11 responders. Thirteen e-miRNAs predicted IMM failure with 90% sensitivity and 100% specificity. Ingenuity pathway analysis (IPA) determined 10 of the 13 e-miRNAs were significantly associated with angiogenesis-related biological functions (p < 0.025) and angiogenic factors that have been associated with recurrent ischemic events in ICAD. These e-miRNAs included miR-122-5p, miR-192-5p, miR-27b-3p, miR-16-5p, miR-486-5p, miR-30c-5p, miR-10b-5p, miR-10a-5p, miR-101-3p, and miR-24-3p. As predicted by IPA, the specific expression profiles of these 10 e-miRNAs in non-responders had a net result of inhibition of the angiogenesis-related functions and up expression of the antiangiogenic factors. This study revealed distinct expression profiles of circulating e-miRNAs in refractory ICAD, suggesting an antiangiogenic mechanism underlying IMM failure.

## Introduction

Intracranial atherosclerotic disease (ICAD) is one of the most common causes of stroke worldwide^1–3^. It accounts for at least 10% of all strokes in the United States^4^ and as much as 50% in countries with predominantly Asian, Hispanic, and Black populations, which constitute the majority of the world^3^. Randomized controlled clinical trials have shown that angioplasty with stenting and bypass surgery fail to improve outcomes in patients with ICAD^5–7^, and that intensive medical management (IMM) achieves the best results. However, despite IMM, ICAD carries a worse prognosis than other stroke etiologies, with an annual rate of recurrent stroke and death of 15%^8^ and as high as 35% in certain populations^9,10^, which highlights the need to develop a better understanding of the molecular mechanisms underlying the disease progression and new targets for effective therapies.

Recent studies suggest a potential regulatory function for several microRNAs (miRNAs) on inflammation, endothelial dysfunction, and vascular smooth muscle cell differentiation contributing to the evolution of atherosclerotic cervical carotid plaque toward growth, instability, and rupture^11,12^. Nonetheless, the remarkable differences on the structure of the arteries (e.g. endothelial specialized pleiotropic functions, adventia and media thickness, and nature of vasa vasorum) and the epidemiologic risk factors for atherosclerosis between extracranial carotid and intracranial large arteries may limit the application of those previous findings into ICAD^13–15^. A discovery-based approach to a circulating miRNA profile in peripheral blood, as a noninvasive biomarker of ICAD progression and IMM failure, has not yet been investigated.

The importance of exosomes in intercellular communication has been long recognized due to their ability to transfer biological cargoes including miRNA^16^. The circulating exosomes mainly derived from the endothelium, platelet, and blood cells not only represent an encapsulation that protects miRNA from external ribonucleases during transportation, but also enrich the majority of the detectable miRNAs in plasma to a more concentrated level and resultant stronger biological effect^17^. In these terms, the circulating exosomal miRNA (e-miRNA) profile could be an optimal candidate for recognition of high-risk ICAD patients refractory to IMM. Therefore, we prospectively enrolled patients diagnosed with ICAD undergoing IMM and measured the circulating e-miRNA levels at baseline and analyzed the association between e-miRNA expression profiles and recurrent ischemic events during a 6-month follow up. The biological functions and molecular targets of the outcome-related e-miRNA profiles were further investigated in the Ingenuity Pathway Analysis (IPA) environment to gain insight on the mechanisms underlying the disease progression and IMM failure in ICAD.

## Results

### Study subjects data

Seventy-four subjects in total were enrolled in the study. Twenty-nine patients had recurrent ischemic events despite IMM during 6-month follow up (non-responders) and 45 patients had no ischemic events (responders). Using propensity score matching with a tolerance of 0.05, 11 non-responders were matched with 11 responders, considering age, gender, and compliance to IMM. One non-responder was excluded because of insufficient small RNA extraction from the circulating exosomes for miRNA sequencing. The circulating e-miRNA levels of the remaining twenty-one baseline blood samples were measured and processed. The demographic and clinical information of the non-responders and the responders was summarized in Table 1, and only race showed significant difference between the two groups. Patients’ compliance to IMM was evaluated at baseline, 1 month, and 6 months after enrollment. The data of reported compliance and observed goal achievement for the control of stroke risk factors were shown in Table 2, which indicates the patients were compliant with IMM and achieved treatment goals consistently along their follow up. Of the 10 non-responders only 1 patient had a TIA and 8 patients that suffered strokes had borderzone or cortical borderzone ischemia. The angiograhphic collateral scores, using the American Society of Intervention and Therapeutic Neuroradiology/Society of Interventional Radiology (ASITN/SIR) grades were below 2 (no collateral or slow collaterals to the periphery of ischemic site with persistence of some of the defect) for all non-responders, indicating poor collaterals.

**Table 1:** Demographic and clinical characteristics in responders and non-responders.

Variable	Responders (n = 11, %)	Non-Responders (n = 10, %)	P
Age (mean ± SD)	59.1 ± 12.1	57.7 ± 14.0	NS
Gender			NS
Male	4 (36.4)	3 (30.0)	
Female	7 (63.6)	7 (70.0)	
Race			0.03
White	6 (54.6)	2 (20.0)	
Black	3 (27.3)	1 (10.0)	
Asian	1 (9.1)	7 (70.0)	
Native American	1 (9.1)	0 (0.0)	
Lesion Location			NS
MCA	5 (45.5)	5 (50.0)	
ICA	5 (45.5)	5 (50.0)	
Basilar	1 (9.0)	0 (0.0)	
Previous Stroke	7 (63.6)	6 (60.0)	NS
Smoking History^a^	1 (9.1)	2 (20.0)	NS
Hypertension	9 (81.8)	5 (50.0)	NS
Hypercholesterolemia	3 (27.3)	3 (30.0)	NS
Hypertriglyceridemia	3 (27.3)	4 (40.0)	NS
Diabetes Mellitus^b^	4 (36.4)	3 (30.0)	NS
Obesity	2 (18.2)	2 (20.0)	NS
Coronary Artery Disease	1 (9.1)	2 (20.0)	NS
Peripheral Artery Disease	0 (0.0)	0 (0.0)	NS
Chronic Renal Disease	1 (9.1)	1 (10.0)	NS
Neoplasm	0 (0.0)	0 (0.0)	NS

^a^An adult who had smoked 100 cigarettes in his or her lifetime was considered with smoking history.

^b^All patients were diagnosed as type 2 diabetes mellitus.

MCA, middle cerebral artery; ICA, intracranial cerebral artery; IMM, intensive medical management; NS, no significance; SD, standard deviation.

**Table 2:** Reported proportion of IMM compliance (%) and observed proportion of IMM target achievement (%) in responders and non-responders.

Response	Baseline		1 month		6 months	
	Yes	No	Yes	No	Yes	No
IMM compliance						
antiplatelets	100.0	100.0	90.9	90.0	100.0	100.0
statins	100.0	100.0	100.0	100.0	100.0	100.0
BP medications	100.0	100.0	100.0	100.0	100.0	100.0
DM treatment	100.0	100.0	100.0	100.0	81.8	80.0
IMM target achievement^a^						
LDL ≤ 70 mg/dL	54.5	50.0	72.7	60.0	54.5	
SBP ≤ 150 mmHg	63.6	70.0	54.5	60.0	63.6	
HbA1c ≤ 7%	63.6	70.0	72.7	80.0	72.7	
vigorous physical activity	27.3	20.0	36.3	40.0	54.5	
smoking cessation	72.7	80.0	81.8	100.0	100.0	

^a^The target achievement was evaluated until any recurrence of ischemic event.

IMM, intensive medical management; BP, blood pressure; DM, diabetes mellitus; LDL, low-density lipoprotein; SBP, systolic blood pressure; HbA1c, glycated hemoglobin A1c.

### E-miRNA expression profile according to the recurrent ischemic event despite IMM

**Figure 1:**
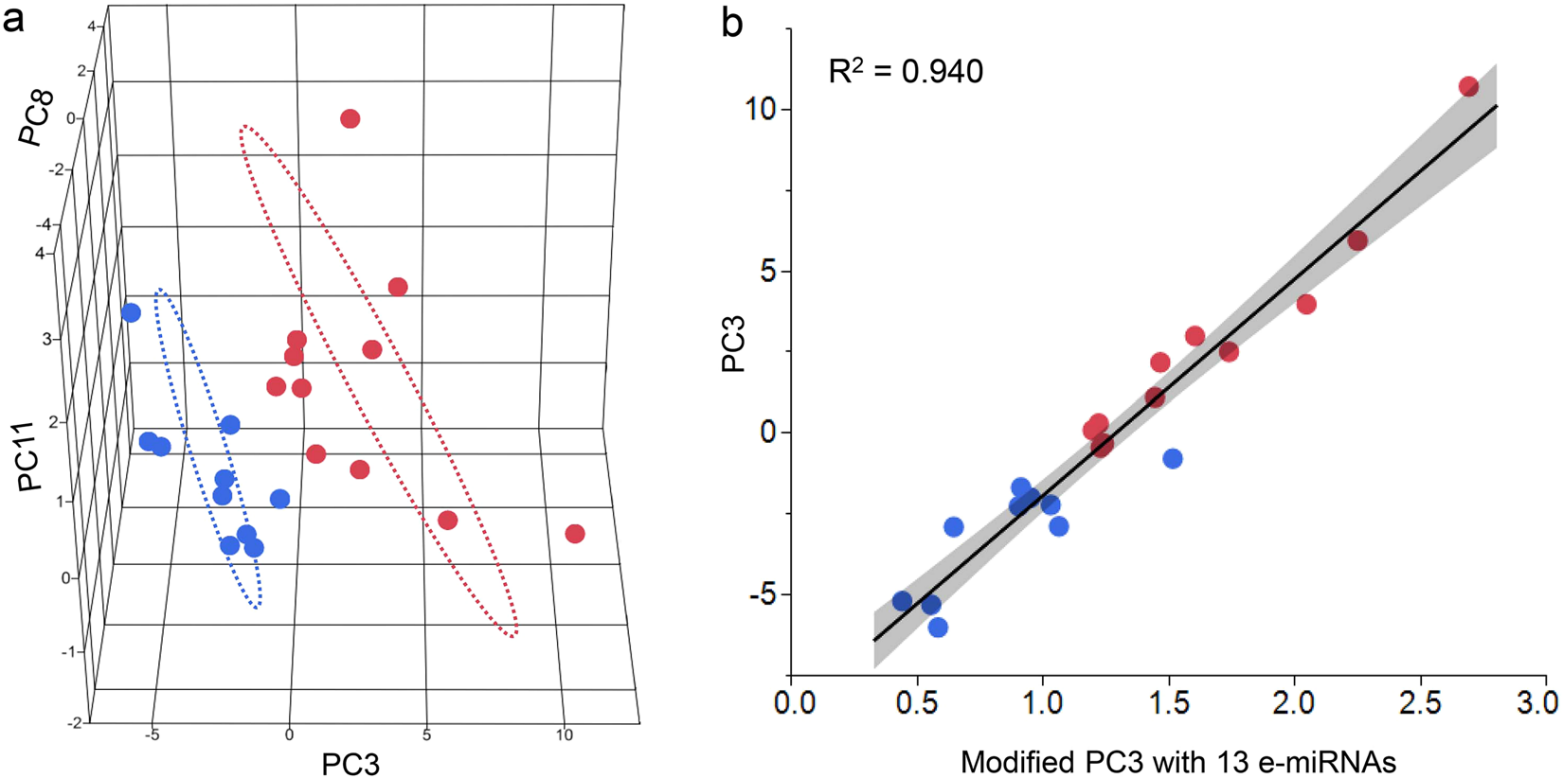
Principal component analysis (PCA) of the e-miRNA expression profiles in severe ICAD patients and correlation analysis between PC3 and 13 e-miRNAs. 3D Scatterplot of PCA of the e-miRNA expression profiles in severe ICAD patients (panel a). The principal component 3 (PC3) was statistically significantly associated to the outcome of response to medical management (p = 0.0052). When PC3 is used on the horizontal X axis, there is an evident differentiation in the distribution of the e-miRNA expression between ICAD patients with recurrent ischemic events despite IMM (blue dots) and those responding to medical management (red dots). Panel b shows the correlation analysis between PC3 and the modified PC3 computed by using only the 13 e-miRNAs with the highest absolute coefficients (>75% quantile). There is a preserved high correlation between the original PC3 and the modified PC3 (R^2^ = 0.94) and the modified PC3 is able to differentiate non-responders to IMM (blue dots) from responders (red dots).

**Figure 2:**
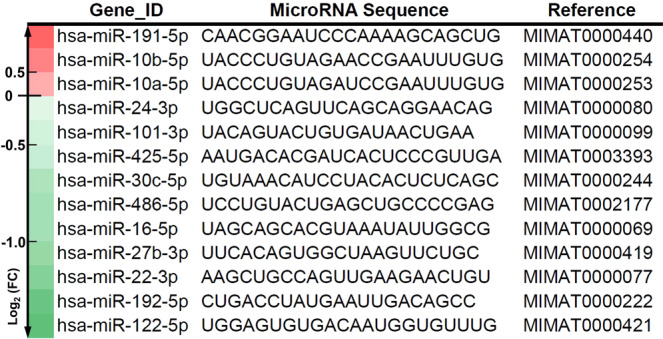
Identification, sequence, and reference number of e-miRNAs. Identification, sequence, and reference number of the 13 e-miRNAs with the highest absolute coefficients in principal component 3 with a color scale quantified by log_2_ fold change (FC) on the left, representing the degree of the upexpression (in red) and the downexpression (in green) of these e-miRNAs in the blood samples from the non-responders

A total of 122 e-miRNAs were mapped to reference genome and isolated after noise elimination. The expression data of these e-miRNAs were used to conduct the principle component analysis (PCA), which generated 13 principle components (PCs) with eigenvalues higher than 1, accounting for more than 95% of the data variation. These PCs were named “PC1” to “PC13” in order of decreasing eigenvalue. PC3 was the only component that was significantly associated with phenotypic outcomes of responders and non-responders (p = 0.0052, Fig. 1a). A receiver operative curve analysis demonstrated the predictive ability of PC3 for recurrent ischemic event despite IMM with 100% sensitivity and 100% specificity. Thirteen significant e-miRNAs with the absolute coefficients above 75% quantile were identified as the major contributors of the PC3, including miR-27b-3p, miR-122-5p, miR-16-5p, miR-30c-5p, miR-486-5p, miR-10a-5p, miR-10b-5p, miR-101-3p, miR-24-3p, miR-192-5p, miR-30c-5p, miR-425-5p, and miR-191-5p. The modified PC3 with only these 13 e-miRNAs as components was highly correlated with the original PC3 (R^2^ = 0.940) and able to predict recurrent ischemic event despite IMM with 90% sensitivity and 100% specificity (Fig. 1b). The specific expression changes of these meaningful e-miRNAs in non-responders is shown in Fig. 2.

### Associations of e-miRNAs expression profile with biological functions and molecules

**Figure 3:**
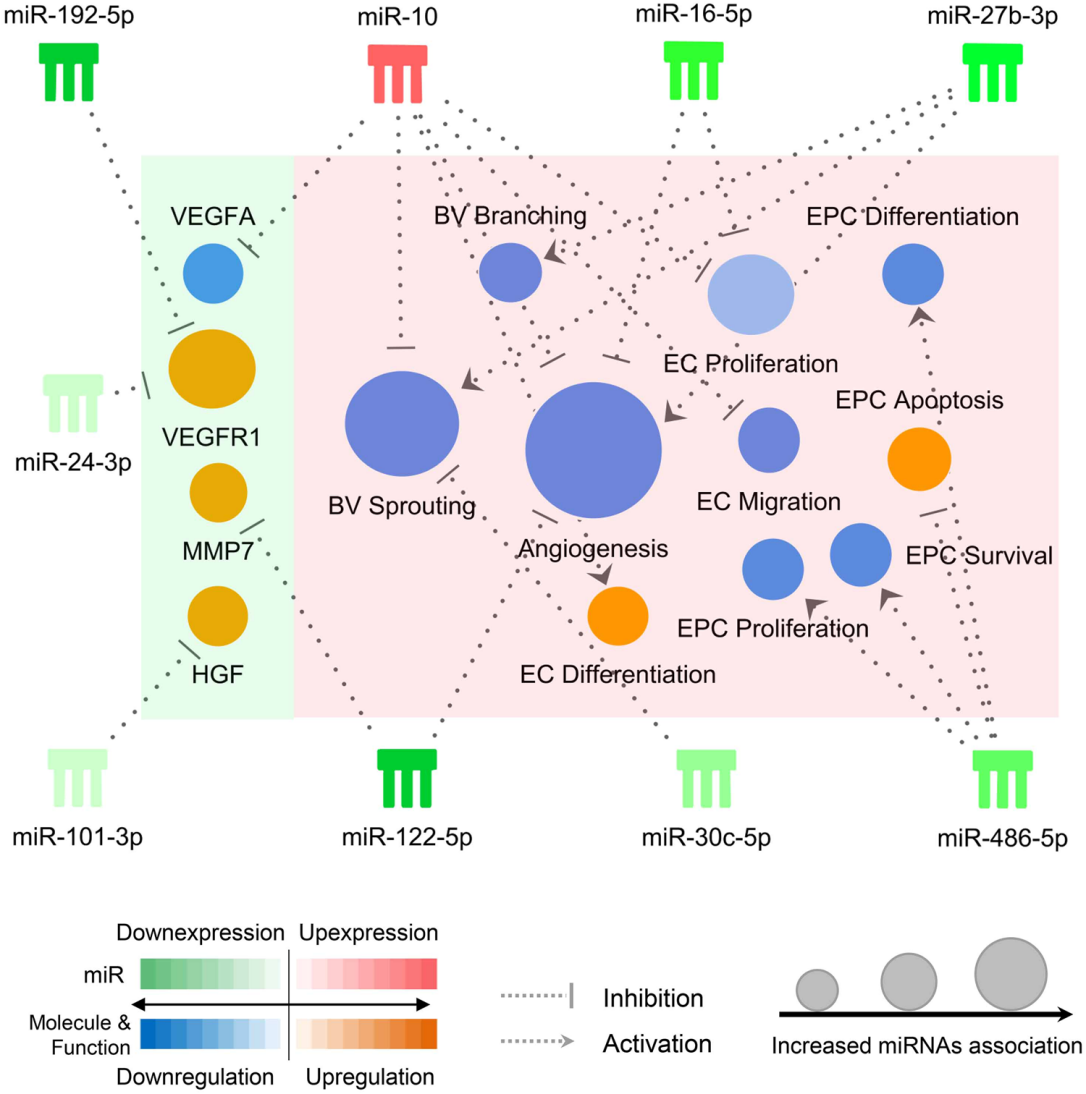
Schematic illustration of a causal network in the Ingenuity Pathway Analysis (IPA) environment. The figure shows the dominant e-miRNAs (comb-shaped symbols), their significantly associated angiogenesis functions (circles in the pink shade) and angiogenic factors (circles in the cyan shade). The angiogenic factors selected (VEGFA, VEGFR1, MMP7, and HGF) were associated to recurrent ischemic events in ICAD in previously published studies (Reference #21 and #22). The e-miRNAs and their target molecules and functions are connected through dotted lines ended with either an arrow or a perpendicular solid line, indicating activated or inhibitory effect, respectively. A biological function or molecule with a stronger association to e-miRNAs has a larger circular size. The relative expression levels of the e-miRNAs in non-responders were scaled by a diverging palette from dark green (downexpression) to dark red (upexpression), while the regulatory effect on the biological functions and molecules were scaled by the other diverging palette from dark blue (downregulation) to dark orange (upregulation). The specific e-miRNA expression profiles in non-responders is predicted by the molecular activity predictor (MAP) to inhibit all the proangiogenesis-related biological functions with the exception of differentiation of endothelial cells, resulting in a net inhibition of angiogenesis. The expression change of the molecules in accordance to the e-miRNA expression profile in non-responders is consistent with the actual findings in the previous studies. VEGFA, vascular endothelial growth factor A; VEGFR1, vascular endothelial growth factor receptor 1; MMP7, matrix metalloproteinase 7; HGF, hepatocyte growth factor; BV, blood vessel; EC, endothelial cell; EPC, endothelial progenitor cell; miR, microRNA

The 13 e-miRNAs considered as the major contributors of the PC3 were analyzed in the Ingenuity Pathways Analysis (IPA) environment. Seven of the 13 meaningful e-miRNAs, consisting of miR-27b-3p, miR-122-5p, miR-16-5p, miR-486-5p, miR-30c-5p, and miR-10 as the notation of the family of miR-10a-5p and miR-10b-5p had in the functional analysis statistically significant associations with angiogenesis-related functions, which includes angiogenesis (p = 0.0038), sprouting (p = 0.0020) and branching (p = 0.022) of blood vessels, proliferation (p = 0.020), migration (p = 0.025), and differentiation (p = 0.042) of endothelial cells, and apoptosis (p = 0.0059), survival (p = 0.0026), proliferation (p = 0.0051), and differentiation (p = 0.0095) of CD34^+^ cells. Besides, miR-30c-5p was associated with quantity of atherosclerotic lesions (p = 0.0062). MicroRNA target analysis demonstrated that miR-101-3p, miR-122-5p, miR-24-3p, miR-192-5p, and miR-10 were upstream modulator of VEGFA, VEGFR1, HGF, and MMP7 as a main proteinase for production of angiostatin and endostatin, which have been shown association with recurrent ischemic events in ICAD by the previous studies (Fig. 3). Furthermore, the regulatory effects of the distinctive e-miRNA expression profile in non-responders on the associated angiogenesis-related functions and molecules were predicted via molecular activity predictor (MAP) tool, which revealed a net effect of inhibition of all the angiogenesis-related functions. In addition, up-regulation of matrix metalloproteinase 7 (MMP7), vascular endothelial growth factor receptor 1 (VEGFR1), and hepatocyte growth factor (HGF) and down-regulation of vascular endothelial growth factor-A (VEGF-A) were observed in correspondence to the miRNA profile in non-responders. A sensitivity analysis to address the potential impact of including TIA in the primary endpoint demonstrated that there was no difference in the direction of up or down regulation of any of the seven e-miRNAs included in the IPA analysis when only patients with stroke were considered as non-responders. Furthermore, the analysis demonstrated a larger difference between responders and non-responders in the levels of miR-27b-3p, miR-486-5p, miR-30c-5p, miR-10a-5p, and miR-10b-5p.

## Discussion

The present study is the first survey of intracranial atherosclerosis patients receiving IMM to associate the e-miRNA expression profile with recurrent ischemic events despite treatment. The results identified a differential expression of e-miRNA between responders and non-responders. Furthermore, the distinctive e-miRNA expression profile indicated antiangiogenic mechanisms underlying the disease progression and IMM failure.

In a total of 74 enrolled patients receiving IMM, 29 (39.1%) of them experienced ischemic events during 6-months follow up in the current study. The proportion of non-responders is higher than expected, which is partially attributed to the inclusion of TIA as a criterion of IMM failure. However, the occurrence of transient ischemia indicates the evolution of atherosclerosis and inefficacy of IMM and suggests upcoming stroke that may cause severe disability and death. As a result, the critical fact that a high proportion of cases are refractory to the optimal medical treatment impose an urgent challenge to discern the non-responders in a timely and precise manner. To our best knowledge of literature, two studies have been performed for this purpose, from the perspective of angiogensis mechanisms. Arenillas *et al*. preliminarily evaluated the proangiogenic VEGF and the antiangiogenic endostatin in 40 patients with ICAD. They found that a higher endostatin/VEGF ratio and an elevated endostatin level were associated with higher intracranial atherosclerotic burden and risk of recurrent stroke, respectively^18^. A latest research conducted by Gonzalez *et al*. expanded their investigation into 21 circulating pro and antiangiogenic factors and observed up-regulation of angiostatin, endostatin, VEGFR1, and HGF and down-regulation of VEGF-A increasing the risk of IMM failure and corresponding recurrent ischemic events^19^. Both of the former studies suggest a key role of antiangiogenesis in ICAD with recurrent ischemic events. Interestingly, the current study on a different molecular type of biomarker also points to the antiangiogenic mechanisms underlying the disease progression. Furthermore, the predicted expressions of MMP7 (a main proteinase for production of endostatin and angiostatin^20,21^), VEGFR1, HGF, and VEGF-A based on the e-miRNA expression profile observed in the study and miRNA-protein regulation established by IPA is completely consistent with the actual findings in the above-mentioned research, which increases the confidence of this relationship.

Although our results suggest an intriguing role of the significant e-miRNAs in the antiangiogenic mechanism and recurrent ischemic events, the factors that induce the over- and under-production of these specific e-miRNAs remain unclear. As all of these e-miRNAs were relatively abundant in both groups of responders and non-responders, genetic mutation and epigenetic silence that commonly lead to the absent mapping of the affected miRNAs are less likely to be the cause. Conversely, the environmental factors may contribute more to the differential expression. A previous study demonstrated that profound changes of miRNA expression including increased miR-10 and miR-27b could be induced by laminar shear stress, which has been related to a potent atheroprotective effect^22^. Metabolic syndrome, as a vascular risk factor, has also been linked to the miRNA expression. Hyperglycemic repression of miR-16 and miR-24 were found in experimental models of diabetes mellitus^23,24^, and a sustained long-chain polyunsaturated fatty diet could lead to the increased level of circulating miR-192 and miR-486-5p in healthy subjects. As platelets are one of the main sources of the circulating miRNAs, the association between miRNA expression and platelet activation is of interest to investigate. A recent research focusing on this point showed that plasma level of miR-191 was decreasing along with the increasing dose of anti-platelet drugs^25^. In these terms, the specific e-miRNA expression profile of the non-responders in the current study could correspond to a more disrupted laminar flow, severer hyperglycemia, less polyunsaturated fatty diet assumption, and lower anti-platelet status, which is presumably consistent with higher risk of ischemic events.

Regarding the two former studies investigating the association of miRNA with carotid plaque destabilization, Maitrias *et al*. compared the tissue expression of seven miRNAs allegedly involved in plaque growth and instability and found miR-100, miR-125a, miR-127, miR-133a, miR-145, and miR-221 levels were markedly different between symptomatic and asymptomatic plaques^11^. A more recent study compared circulating e-miRNAs in patients between stable stenosis and progressive stenosis of internal carotid artery. A significant higher expression of miR-199b-3p, miR-130a-3p, miR-24-3p, miR-221-3p, and miR-27b-3p were observed in patients with stenosis progression^12^. The inconsistent results among the three studies including the current one may be explained by the dissimilar study designs including the source of miRNA (tissue vs. peripheral blood and exosomal vs. free) and related outcome (stenosis progression vs. ischemic event). The difference also suggests the distinct pathophysiology of atherosclerosis progression in various location.

Some limitations of this study require recognition. Although a sophisticated matching algorithm has been undertaken to decrease the demographic imbalance between groups and to minimize confounding of outcome-related clinical covariates on the miRNA expression, race could not be matched between responders and non-responders. Since race has been reported to be associated with miRNA expression, the racial impact on the distinctive e-miRNA expression profiles between two groups could not be clearly eliminated. However, to the best of our knowledge, only two of the ten miRNAs that were associated with angiogenic factors and angiogenesis-related biological functions have been found to be affected by on race in the literature, including miR-30c^26^, and miR-24^27^; and both miRNAs have been shown differential expressed between African Americans and Caucasian Americans, instead of between Asians and Caucasians, which was the main difference in the current study.  Therefore, a race-matched study will be  necessary to further verify our findings. The use of TIA as an endpoint is controversial. However, the post hoc sensitivity analysis to address the potential impact of including TIA demonstrated no differences in the up or down regulation of any of the seven e-miRNAs analyzed in the IPA environment, when only stroke cases were considered. An additional limitation is that the sample size of the research is relatively small. To reduce the false discovery rate, several strategies were applied including PCA to avoid exhaustive inferential comparisons and focus the inferential analysis on the most suitable candidates, and Bonferroni corrections to establish the levels of significance. We acknowledged the exploratory aim of this pilot study without validation cohort and suggestive results computed by IPA. However, it is important to recognize that the study of miRNA-gene interaction and miRNA-function relevance can be laborious and time consuming due to the difficulty of experimental model establishment^28^. The application of causal network analysis on the foundation of the peer-reviewed literatures in IPA is able to eliminate the bias due to inadequate literature mining and incomprehensive perception and thus provide a conscientious and precise insight.

In summary, our study suggests a specific circulating e-miRNA expression profile that is associated with antiangiogenesis and is a novel biomarker for recurrent ischemic events despite IMM in severe ICAD. Further studies in a larger cohort of patients is necessary to confirm our findings.

## Methods

### Subjects and study design

In this prospective cohort study, patients aged ≥ 30 years diagnosed as severe ICAD (≥70% stenosis) of any intracranial large artery in four hospitals in Los Angeles metropolitan area between 2012 and 2016 were considered for inclusion. Independent vascular neurologists determined patient eligibility, based on the inclusion and exclusion criteria in Table 3. Immediately upon enrollment, all patients underwent IMM for primary risk factors (systolic blood pressure [SBP] and low-density lipoprotein [LDL]), secondary risk factors (diabetes, smoking, obesity, and inadequate exercise) and antiplatelet medications. Blood samples were collected in EDTA tubes at baseline, and then transported to the laboratory of each hospital within 10 minutes. Plasma was immediately separated by centrifugation at 3500 rpm for 15 minutes at 4 °C, aliquoted in 2 cc cryogenic vials, and stored at −80 °C for subsequent e-miRNA extraction and sequencing.

**Table 3:** Inclusion and Exclusion Criteria.

Inclusion criteria	Exclusion criteria
1. ICAD with 70% to 99% stenosis of a major intracranial artery diagnosed by angiogram, TCD, MRA, or CTA. 2. Patient is willing and able to return for all follow-up visits required by the protocol. 3. Patient understands the purpose and requirements of the study, can make him- or herself understood, and has provided consent.	1. Intracranial arterial stenosis related to arterial dissection, moyamoya disease, or any known infectious or vasculitic disease. 2. Presence of any unequivocal cardiac sources of embolism. 3. Any hemorrhagic infarct within 14 days before enrollment or any other intracranial hemorrhage (subarachnoid, subdural, or epidural) within 30 days. 4. Endovascular angioplasty and (or) stenting or major surgery (including open femoral, aortic, or carotid surgery) within previous 30 days or planned in the next 180 days after enrollment. 5. Intracranial tumor or vascular malformation. 6. Severe neurologic deficit that renders the patient not independent.

ICAD, intracranial atherosclerotic disease; TCD, transcranial Doppler ultrasound; MRA, magnetic resonance angiography; CTA, computed tomography angiography.

After initiation of IMM, all patients received coaching with monthly phone calls that went over their medications, smoke cessation, physical activity engagement, and weight control. Additionally, patients undertook follow-up visits in person at one, three, and six months after enrollment. At one-month and six-month follow-up visits, a vascular neurologist not involved in the data analysis evaluated the patient’s reported compliance to IMM and the measurements of SBP, laboratory results of LDL and Hemoglobin A1c, and inquiry of smoking and physical activity for assessment of the control of the primary and secondary risk factors. In addition, any occurrence of TIA or ischemic stroke after enrollment was determined at each visit and considered as recurrent ischemic events and IMM failure. Of note, all the ischemic event should be clearly defined by the stroke neurologists as secondary to ischemia and no other causes such as seizures or recrudescence of initial symptoms due to systemic reasons such as febrile states, infections, or fatigue. In addition, when only a TIA was the symptom, required a clear correlation between neurological deficits and hypoperfusion. The research coordination center monitored patient adherence to the scheduled visits, and transport services were provided to encourage patient adherence to the protocol.

**Figure 4:**
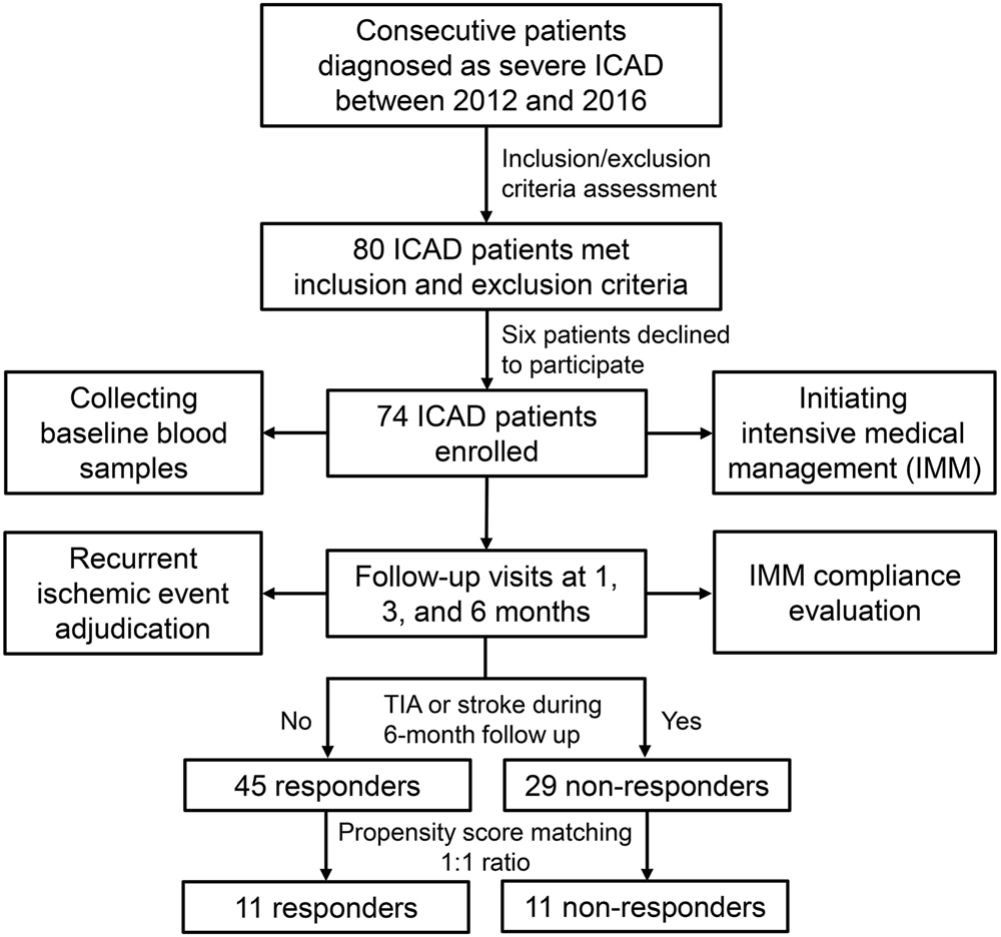
Flow chart of the cohort study combined with propensity score matching.

The patients were divided into two groups, termed as non-responders and responders, based on the occurrence of any TIA or stroke despite IMM during 6-month follow up. Then, propensity score matching with a tolerance of 0.05 was applied to match the patients from each group with 1:1 ratio, considering age, gender, and compliance to medical management. A flowchart of the progress through the study was shown in Fig. 4. This study received approval from the institutional review boards of Cedars-Sinai Medical Center and the University of California, Los Angeles and informed consent was obtained from each participant. All experiments were performed in accordance with relevant named guidelines and regulations.

### Exosome extraction and purification

Plasma sample with the volume of 500 μl was centrifuged at 1500 rpm for 5 min to remove residual cells and debris. The supernatant was transferred to a new 1.5 ml Eppendorf tube for exosome isolation. ExoQuick (Cat# EXOQ5A-1, System Biosciences, Palo Alto, CA) was added to the supernatant at 1:4 ratio (ExoQuick:Supernatant), mixed gently, and allowed to incubate for 30 min at 4 °C. After the incubation, the admixture was centrifuged at 1500 rpm for additional 30 min to recover the exosomes. Using ExoQuick-based precipitation techniques for exosome isolation and recovery is broadly recognized for better specific exosomal miRNAs recovery and lower intra-assay coefficients of variability (CVs) compared to other commercial exosome isolation kits, and is also backed in nearly 600 peer-review citations^29^.

### Exosome total RNA isolation

Exosomes extracted from the samples were processed for total RNA isolation using the SeraMir Exosome RNA Purification Column kit (Cat #RA808A-1, System Biosciences, Palo Alto, CA) according to the manufacturer’s instructions. For each sample, 1 µl of the final RNA eluate was used for measurement of small RNA concentration by Agilent Bioanalyzer Small RNA Assay using Bioanalyzer 2100 Expert instrument (Agilent Technologies, Santa Clara, CA). A small RNA concentration of 200 pg/ul was considers as a cutoff for subsequent library preparation.

### Next generation sequencing (NGS) library generation and e-miRNA sequencing

Small RNA libraries were constructed with the CleanTag Small RNA Library Preparation Kit (Cat# L-3206, TriLink BioTechnologies, San Diego, CA) according to the manufacturer’s protocol. The final purified library was quantified with High Sensitivity DNA Reagents (PO# G2933-85004, Agilent Technologies, Santa Clara, CA) and High Sensitivity DNA Chips (PO# 5067–4626, Agilent Technologies, Santa Clara, CA). The libraries were pooled, and the 140 bp to 300 bp region was size selected on an 8% TBE gel (Ref# EC6215, Invitrogen by Life Technologies, Grand Island, NY). The size selected library was quantified with High Sensitivity DNA 1000 Screen Tape (PO # 5067–5584, Agilent Technologies, Santa Clara, CA), High Sensitivity D1000 reagents (PO# 5067–5585, Agilent Technologies, Santa Clara, CA), and the TailorMix HT1 qPCR assay (Cat# TM-505, SeqMatic, Union City, CA), followed by a NextSeq High Output single-end sequencing run at SR75 using NextSeq 500/550 High Output v2 kit (Cat #FC-404-2005, Illumina, San Diego, CA) according to the manufacturer’s instructions. Sequencing data were analyzed for quality and contamination using FastQC. Then, the open-source tools including FastqMcf, part of the EA-utils package, and PRINSEQ were used to detect and remove N’s at the ends of reads, trim sequencing adapters, and filter reads for quality and length. The improved set of sequence reads were mapped to the reference genome using Bowtie and expression levels for exosomal miRNAs were calculated using R statistical environment.

### Statistical analysis

The circulating e-miRNA expression levels of the baseline blood samples of the matched subjects were measured and processed through the workflow mentioned above. A cut-off value of 500 was set as a minimal valid read counts per miRNA to eliminate the noise that had been produced in sequencing. Expression data were further normalized using the DESeq method^30^.

Principal component analysis (PCA) was performed to reduce data dimensionality. The principal components (PCs) accounting for 95% of the total variance of the data were selected and calculated for each patient. The association between the PCs and recurrent ischemic events were evaluated via Mann-Whitney tests with Bonferroni correction to determine the predictive PC for recurrent ischemic events and reduce the false discovery rate; binary logistic model was then generated to calculate the sensitivity and specificity via a receiver operative curve (ROC). To further scale the contribution of each e-miRNA to the predictive PC, the absolute coefficients of all the e-miRNAs in the PC formula were distributed in a quartile box plot, and the e-miRNAs with the coefficients above 75% quartile were selected. To interpret the regulatory effect of these selected e-miRNAs, functional analysis and miRNA target analysis was carried out in Ingenuity Pathway Analysis (IPA) environment to mine the significantly associated biological functions and downstream molecules. The up- and down-regulation of the specific e-miRNA expression profile in the non-responders on the relevant biological functions and molecules were predicted via a build-in Molecular Activity Predictor (MAP) tool. The networks, functional analyses, and miRNA target analyses were generated through the use of IPA (QIAGEN Inc, https://www.qiagenbio-informatics.com/products/ingenuity-pathway-analysis). A sensitivity analysis was conducted to address the potential impact of including TIA in the primary endpoint. The expression levels of the relevant e-miRNAs, identified as described above, were compared between the whole cohort and including exclusively subjects with stroke.

The statistical analyses including PCA and binary logistic regression analysis were performed in JMP (version 13). A two-tailed p value of <0.05 was considered statistically significant. The statistical significance of the analysis in IPA environment was calculated by causal analytics algorithms which are based on a “master” network derived from the Ingenuity Knowledge Base^31^.

## Data Availability

The datasets analyzed during the current study are available from NCBI GEO database with the accession number GSE178500.
