# Author Correction: Differential expression of circulating exosomal microRNAs in refractory intracranial atherosclerosis associated with antiangiogenesis

**DOI:** 10.1038/s41598-021-94233-1

**Published:** 2021-07-21

**Authors:** Hao Jiang, Juan F. Toscano, Shlee S. Song, Konrad H. Schlick, Oana M. Dumitrascu, Jianwei Pan, Patrick D. Lyden, Jeffrey L. Saver, Nestor R. Gonzalez

**Affiliations:** 1 Department of Neurosurgery, Neurovascular Laboratory, Cedars-Sinai Medical Center, Los Angeles, CA USA; 2 Department of Neurosurgery, The First Affiliated Hospital of Zhejiang University, School of Medicine, Hangzhou, China; 3 Department of Neurology, Cedars-Sinai Medical Center, Los Angeles, CA USA; 4 Department of Neurology, University of California, Los Angeles, Los Angeles, CA USA

Correction to: *Scientific Reports* 10.1038/s41598-019-54542-y, published online 19 December 2019

The original version of this Article contained an error in the Data Availability section, which did not contain the accession codes for the data. This information is now included and in Data Availability,

"The datasets analyzed during the current study are available from the corresponding author on reasonable request."

now reads

"The datasets analyzed during the current study are available from NCBI GEO database with the accession number GSE178500."

The original Article has been corrected.

